# Conference Report: Federal Wireless Users’ Forum Workshop Gaithersburg, MD June 7–9, 1994

**DOI:** 10.6028/jres.100.008

**Published:** 1995

**Authors:** Mary K. Ruhl, Tish Antonishek

**Affiliations:** Advanced Systems Division, National Institute of Standards and Technology, Gaithersburg, MD 20899-0001

## 1. Introduction

This paper summarizes the fourth workshop of the Federal Wireless Users’ Forum (FWUF) held at the National Institute of Standards and Technology on June 7–9, 1994. FWUF workshops provide the opportunity for potential and current wireless users to learn more about wireless communications technology and issues, define wireless application requirements, and interact with industry and other user participants on wireless requirements, technology, and issues. A highlight of this workshop was the Federal Wireless Policy Committee Industry Outreach Session, in which a dialogue took place between the Federal Government and industry on Federal wireless requirements. Breakout sessions in the areas of Medical Services, Mobile Workplace, Transportation Management, and Soldier’s Radio were held to discuss and document user application requirements.

## 2. Background

The fourth workshop of the Federal Wireless Users’ Forum (FWUF) was held on June 7–9, 1994 at the National Institute of Standards and Technology in Gaithersburg, MD. The workshop was sponsored by the Wireless Services Program Office of the National Communications System (NCS), FWUF, and NIST. The objectives of the FWUF are to:

- Educate Federal Government users about wireless telecommunications;
- Identify the wireless telecommunication needs of Federal Government users;
- Facilitate information exchange with other user groups, standards organizations, manufacturers, and service providers to ensure that Government user wireless needs are met and;
- Support the interoperability of emerging wireless services and equipment through increased participation in the formation of Federal policy, participation in wireless standards development, and other appropriate activities.

While FWUF workshops focus on dealing with wireless communications issues affecting Federal Government users, other wireless users from state and local government and the commercial sector, along with manufacturers and service providers, are welcome to participate. The Federal Government and these other users have many similar requirements for wireless applications. Such requirements, along with unique Federal requirements, are addressed at the workshops.

The first FWUF workshop was held in May 1993. Since the first meeting, the workshops have matured and continue to provide valuable opportunities for sharing information and dialogue among government and industry communities. At the first workshop, the following concerns that face Federal wireless users surfaced repeatedly:

- Spectrum management—How do Federal wireless users deal with the lack of universal frequency coordination, access, and signaling?
- Security (including privacy and legal issues)—What are FWUF security requirements and how can they be supported in a wireless environment?
- Interoperability—Considering the diverse range of wireless technology and services, how can the FWUF ensure that wireless systems are sufficiently interoperable with other wireless systems and interoperate with wireline systems?

These core concerns were identified as an initial set of issues to be addressed.

For the purpose of formally focusing Federal Department and Agency attention on issues raised at the FWUF workshop and taking appropriate action, the Federal Wireless Policy Committee (FWPC) was established. The FWPC is a cross section of major government departments and agencies and seeks to address wireless communications issues and ensure that policies concerning a Digital, Ubiquitous, Interoperable, Transparent, and Secure (DUITS) wireless network are developed. The FWPC has stated that DUITS is a general statement of Federal wireless requirements. The FWPC is chaired by Larry Irving of the National Telecommunications and Information Administration (NTIA), and vice-chaired by Dennis Bodson of the NCS. The FWPC consists of four subcommittees:

- Requirements/Standards—chaired by Richard Dean, NSA;
- Security/Privacy—chaired by Ray Kammer, NIST;
- Policy—chaired by Joe Gattuso, NTIA;
- Acquisitions—chaired by Charles Cape, Department of Commerce.

The FWUF is an independent user group that provides input to the FWPC and develops user application requirements which are used as a basis for the FWPC’s work.

The second FWUF workshop, held in September 1993, was focused on the core issues of interoperability, security, and spectrum management. Recommendations from that workshop were directed to various agencies to continue their wireless efforts, and to the FCC to consider DUITS requirements in Federal Government wireless policies. The third FWUF workshop, held in January 1994, directed attention to the National Information Infrastructure, wireless data, and emergency services and priority access. Significant accomplishments of this workshop included articulation of several Government wireless issues and presentation of these issues to industry and standards forums.

## 3. Workshop Program

Against the backdrop of the first three workshops, the fourth workshop took place in June 1994. A total of 202 people from the Federal Government and industry, along with other users, attended the workshop to discuss and address wireless communications issues. The workshop provided both users and industry the opportunity to learn more about wireless technology and capabilities, user requirements, and to express issues confronting government and industry on the use and development of wireless communications.

The first day of the workshop featured an Industry Outreach Event by the Federal Wireless Policy Committee (FWPC). Reports from the FWPC subcommittees’ chairs on Policy, Standards/Requirements, Security/Privacy, and Acquisitions were presented, which provided further details on government wireless requirements. In particular, the FWPC document, “Current and Future Requirements for Federal Wireless Services in the United States,” was presented for discussion with industry. Industry perspectives on these requirements were presented by MCI, Motorola, the PCS Technology Advocacy Group (PTAG), the Personal Communications Industry Association (PCIA), and AT&T. A question and answer session followed. The following list is a summary of the main points expressed during the Industry Outreach Work Session:

- Requirements outlined in the FWPC document were generally embraced by industry as both feasible and desirable features envisioned for emerging wireless services.
- Wireless DUITS networks are possible but it will be some time before all requirements are met. Individual requirements are in different stages of progress and will reach the market at different times.
- Industry must see a market for wireless DUITS products for development of such products to take place.
- Considering the multiplicity of current wireless systems, technology, and providers, a single common air interface is not feasible.
- The Federal Government needs to sell its vision of interoperability in the global market, considering the international operations of the Federal Government.
- Many Federal wireless requirements are the same as commercial market wireless requirements.
- There are some unique Federal requirements (e.g., priority access). The Federal Government must work with industry to ensure such requirements are well understood.
- For the Federal Government to be a driving force in the wireless market, it must fully define its requirements and share them with industry. The Federal Government should also be more active in wireless standards efforts and industry forums.

The FWPC will continue to define Federal wireless communications requirements and communicate them to industry. The requirements put forth by the FWPC are based on user application requirements that are developed at the FWUF workshops.

The morning of the second day of the workshop highlighted updates on the previous workshop topics of National Information Infrastructure (NII), Emergency Services and Priority Access, and Wireless Data. Overviews on Satellite/Terrestrial Interoperability and International Access were presented. On the afternoon of the second day and the morning of the third day, breakout sessions on Medical Services, Mobile Workplace, Soldier’s Radio, and Transportation Management were held. During these sessions, users from numerous agencies discussed common wireless requirements and continue documenting their needs and developing the scope of work. Industry participants were helpful in these discussions by instructing users on the capabilities of current wireless systems. During the afternoon of the third day, a general plenary was held in which the participants of the application breakout groups reported their progress and results.

The FWUF workshops continue to provide valuable opportunities for sharing information and dialogue among government and industry communities. Accomplishments of the fourth workshop include:

- A dialogue between the FWPC and industry on Federal wireless requirements has been established through an Industry Outreach Event.
- Federal user requirements are being carried into wireless standards organizations and industry associations.
- The Federal Government requirements detailed in FWPC requirements document were generally embraced by industry as feasible and desirable features for emerging wireless services.
- Tutorials on wireless communications and issues have been held to educate current and future wireless users.
- Progress has been made on user applications requirements. Defining user application requirements is a primary objective of the FWUF workshops. More participants are needed to fully document the Federal Government requirements.

The fifth workshop of FWUF was recently held at the Hilton Hotel in Gaithersburg, Maryland, on October 5–7, 1994. Topics for the overview sessions were Land Mobile Radio (LMR), Specialized Mobile Radio (SMR), and Enhanced SMR (ESMR), as well as the Intelligent Vehicle Highway System (IVHS). The development of application requirements continued in breakout sessions. Future workshops are currently being planned.

Further information about this program can be obtained by contacting the authors via Internet at mruhl@nist.gov or tantonishek@nist.gov.

